# Evaluation of functional tests performance using a camera-based and machine learning approach

**DOI:** 10.1371/journal.pone.0288279

**Published:** 2023-11-03

**Authors:** Jindřich Adolf, Yoram Segal, Matyáš Turna, Tereza Nováková, Jaromír Doležal, Patrik Kutílek, Jan Hejda, Ofer Hadar, Lenka Lhotská

**Affiliations:** 1 Czech Institute of Informatics, Robotics and Cybernetics, Czech Technical University in Prague, Prague, Czech Republic; 2 BGU Ben-Gurion University of the Negev, Beer Sheva, Israel; 3 Faculty of Physical Education and Sport, Charles University, Prague, Czech Republic; 4 Faculty of Biomedical Engineering, Czech Technical University in Prague, Kladno, Czech Republic; University of Engineering and Technology Taxila Pakistan, PAKISTAN

## Abstract

The objective of this study is to evaluate the performance of functional tests using a camera-based system and machine learning techniques. Specifically, we investigate whether OpenPose and any standard camera can be used to assess the quality of the Single Leg Squat Test and Step Down Test functional tests. We recorded these exercises performed by forty-six healthy subjects, extract motion data, and classify them to expert assessments by three independent physiotherapists using 15 binary parameters. We calculated ranges of movement in Keypoint-pair orientations, joint angles, and relative distances of the monitored segments and used machine learning algorithms to predict the physiotherapists’ assessments. Our results show that the AdaBoost classifier achieved a specificity of 0.8, a sensitivity of 0.68, and an accuracy of 0.7. Our findings suggest that a camera-based system combined with machine learning algorithms can be a simple and inexpensive tool to assess the performance quality of functional tests.

## Introduction

The number of people that need physical therapy increased during the last decades [1]. The cost of rehabilitation treatment increased consequently. The patients are demanding a better patient experience. There is a need for more doctors and assistants together with better services and individualized approaches. Such a system will be unsustainable shortly. During the last decade, advanced technology allowed us to look at the problem from an entirely new perspective and create systems based on the current knowledge and technology level.

For physiotherapists (PTs) or athletic trainers, visual observation is standard practice. The observational analysis relies on the skill of the evaluators and a clinical evaluation that identifies possible deficiencies in movement expression. Performing functional tests and their clinical, subjective evaluation is a common examination method in the differential diagnostics of physicians and physiotherapists [2]. Such tests typically combine screening of a range of motion, strength, and proprioceptive assessment. Examples of such tests are the well-known Single leg squat (SLST) and Step-down tests (SDT). McGovern [3] describes the implementation and possibility of evaluation by one or more experts observing the patient. Schurr et al. [4] proved that for lower extremity movement, 2D analysis is comparable to the frontal plane of 3D motion analysis commonly regarded as the gold standard. However, this only applies if the camera capturing the person is perpendicular to the frontal plane and is positioned at the center of the patient’s body. If an error in the exercise execution can be detected by an expert from a video, we hypothesize that the same error can be detected by machine learning algorithms. Harris-Hayes et al. [5] demonstrated reliability based on visual assessment of lower extremity movement patterns by observing classic 2D RGB recordings.

A PT can use simultaneous motion assessment to assess knee joint dysfunction or pain [6, 7], as well as to assess the hip, pelvic, and trunk deviations, which are also important in people with hip pain [3, 8–10]. The development of modern motion capture (MoCap) systems makes it possible to automatically evaluate the performance of functional tests. Methods for automatic evaluation of similar tests using 3D MoCap techniques have been proposed in the past. Mostaed [11] compared the visually-assessed quality of the step-down test and corresponding Vicon data at [11], Ageberg [12] compared Vicon and Visual analysis for single-limb mini squat. Barker-Davis [13] measured subjects performing the leg squat exercise using Vicon and compared the data obtained with the subjective assessment of five independent experts who evaluated the correctness of the performance by observing videos taken at the same time.

In their comprehensive analysis, Debnath [14] and colleagues provide an extensive review of computer vision-based systems used in physical rehabilitation over the past two decades. The authors propose an innovative taxonomy, categorizing these systems according to the perspective of rehabilitation and assessment. They classify the mode of rehabilitation into two types: Virtual and Direct. In terms of assessment, the authors suggest three different approaches: Comparison, Categorization, and Scoring. Their exhaustive review not only serves as an excellent overview of all applications and approaches within this field but also suggests a unique perspective to classify and understand them. Coyer [15] has compiled a review outlining the state-of-the-art, markerless systems through 2018. This review includes camera-based systems, as well as more hardware-complex systems. Since then, object detection systems based on deep neural networks have mainly been used in the field of camera systems. Currently, the most used open-source systems based on the above methods are OpenPose [16], AlphaPose [17, 18] and Google Mediapipe [19]. They all work on a similar principle and even use similar databases—usually COCO [20] and MPII [21]—for training to create their models. It is important to notice that OpenPose (OP) requires a powerful GPU to process the video, an issue that might disturb the option to use our solution. In our previous work we have demonstrated where to use a more efficient solution without the need for a GPU to process the videos in real-time [22]. In our next study [23], we proved that OpenPose is robust in terms of quality, video resolution, and lighting changes. Thus, its use is in principle not limited by the environment where the measurement is performed. Ota’s study [24] has substantiated the robustness and precision of OpenPose in keypoint extraction, particularly in motion analysis. The investigation utilized OpenPose to study the movements of 20 healthy young participants performing bilateral squats. The joint angles—pertaining to the trunk, hip, knee, and ankle—as calculated by OpenPose were contrasted with those captured by the highly accurate VICON motion analysis device.

Intraclass correlation coefficients (ICCs) indicated an almost flawless consistency between the data generated by OpenPose and VICON, thereby manifesting OpenPose’s high reliability. While minor biases were noted for certain joints, they were documented for future corrections. This investigation verifies not only the reliability of OpenPose but also its cost-effectiveness and user-friendliness compared to traditional methodologies.

Further research validating the reliability of systems like OpenPose using these methodologies has been confirmed across several studies. These include comparisons of 2D and 3D accuracy in actions like lifting [25] and squatting [24].

As demonstrated in all of these studies, it is possible to obtain objective data on the quality of testing by using objective measurement methods. For these functional assessments, complex motion capture systems based on markers and complicated setups are required in a laboratory environment in order to obtain reliable results. Our presented method allows evaluating functional tests directly from video acquisition without the need for complex MoCap systems that use active or passive markers placed on the patient’s body to determine the validity of the test. With this idea, it would be possible to capture the movements of the subjects during the functional tests using ordinary RGB cameras. A system of this type would considerably increase efficiency and reduce the cost of conventional test measurement and evaluation.

Physical therapy is becoming increasingly necessary for many individuals, resulting in rising rehabilitation costs and a demand for better patient experiences. Traditional observational analysis by physiotherapists (PTs) relies on their skills and clinical evaluations to identify deficiencies in movement expression. This study investigates the feasibility of using a camera-based system and machine learning algorithms to assess the quality of functional tests, specifically the Single Leg Squat Test and Step Down Test. Movement analysis can be tailored based on user rating preference behavior models, and OpenPose keypoint extraction can benefit from image-based feature refinement. The AdaBoost classifier’s performance in predicting physiotherapists’ assessments demonstrates the potential of camera-based systems and machine learning algorithms in evaluating functional tests.

Our research has made a significant contribution to the field of functional testing. One of the main contributions of our study is the novel use of only one camera and machine learning algorithms to evaluate functional tests. This approach presents a cost-effective and convenient alternative to current evaluation methods, which often require manual evaluation or the use of expensive devices. This methodology stands in contrast to previous studies conducted by Whelan et al. [26], where wearable gyro-accelerometers were utilized to assess single-leg squats, as well as Mitternacht et al. [27], who employed a similar approach to investigate lower-limb motion characteristics. Furthermore, Seifallahi et al. [28] employed Microsoft Kinect to detect Parkinson’s disease based on skeletal motion parameters. By introducing our markerless methodology, we address the limitations associated with marker-based systems and wearable devices, offering a streamlined and cost-effective means of evaluating functional tests. We hypothesize that a camera-based system utilizing machine learning algorithms can provide a reliable, cost-effective, and accessible solution to assess functional tests, such as the Single Leg Squat Test and Step Down Test. Specifically, we posit that machine learning algorithms can detect errors in exercise execution that a physiotherapist could identify visually.”

## Methods

**Fig 1:**
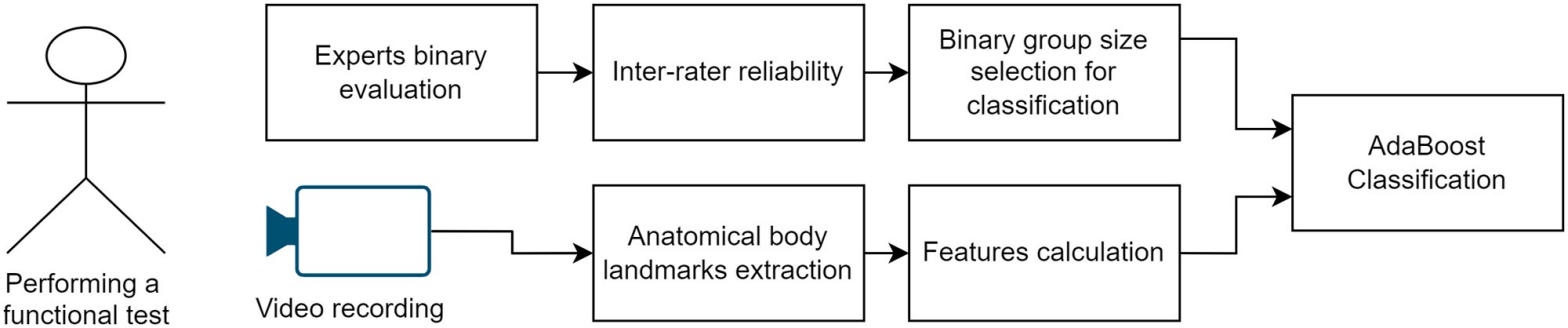
Flowchart of the study design, where we can see the camera acquisition, the expert observing the subjects, and the process till the final system outcome classification.

Our goal was to test the hypothesis that we are able to use a camera-based system to detect the correct execution of functional tests. The sequence of steps we performed to confirm this hypothesis can be seen in Fig 1. The block diagram presented in Fig 1 illustrates a study design combining expert evaluations and video analysis to classify subjects using the AdaBoost algorithm. Channel 1 is used for the training phase, while we use channel two, the patient assessment phase (scoring phase). In Channel 1, experts provide binary assessments for each individual exercise, then we check inter-rater reliability and use them for group size selection. Simultaneously, in Channel 2, the algorithm uses video recordings of subjects; the AI algorithm (OpenPose) extracts anatomical body landmarks (key points), and various features are calculated based on these key points. The outputs from both channels are combined and fed into the AdaBoost Classification algorithm to generate the final system outcome classification. This integrated approach ensures accurate and robust classification, enabling the machine to learn from expert labeling and scores while autonomously performing analysis and measurements of subjects.

### Participants

Forty-six subjects participated in the study: 30 women and 16 men. This was a representative sample of healthy volunteers from a group of students and academic workers from various universities in Prague. The average age was 28.2 ± 7.9 years. The mean height of the subjects was 173.8 ± 9.9 cm, and the most common weight range was 65–70 kg. Half of the subjects identified their right lower limb as dominant, 11 subjects identified their left lower extremity, and 13 subjects had no lateral preference for lower extremity as dominant. Exclusion criteria were any musculoskeletal injury in the past 6 months, acute illness, or pain while performing the test. All participants signed a written informed consent which is institutionally archived. The study was conducted according to the guidelines of the Declaration of Helsinki, and approved by the ethics committee of the Faculty of Physical Education and Sport, Charles University under reference number 167/2020.

### Study design

The study design focused on evaluating participants’ performance in two functional tests, the Single Leg Squat Test (SLST) and the Step-Down Test (SDT), following a standardized procedure. Participants first signed an informed consent form and were provided with information about the purpose and nature of the experiment. They then received video instructions detailing the proper execution of the functional tests to ensure uniform guidance. During the tests, three physiotherapists simultaneously assessed participants’ performance, while a precisely positioned RGB camera recorded the sessions for further analysis. The physiotherapists rated 15 possible execution errors for each test and performed a binary classification for each error (present or absent), providing an objective assessment of the participants’ test performance.

### Functional test parameters and description

Our study examines the performance of two functional tests, the Single Leg Squat Test and Step Down Test. The performance of the used tests followed the protocol developed by Thonnard [2] and McGovern [3]. Following an explanation and demonstration of the test, the subjects were instructed to repeat the test three times for each lower limb. They always returned to an upright standing position between repetitions. Initially, the overall impression must be assessed (balance, gross arm deviation, ability to perform the test), and evaluated as satisfactory to proceed.

At least one of the three repetitions, evaluated as satisfactory, was sufficient to continue the test. The investigator then marked the following criteria as positive or negative:

1. Trunk flexion (forward lean, lateral rotation, lateral flexion, thoracic rotation)
2. Pelvic posture (tilt, rotation)
3. Hip position (adduction, internal rotation)
4. Knee joint position (valgus knee, tremor)
5. Depth of squat (compared to the other side, orientation with T)

Because the tests were not used as clinical screening for screening purposes, the overall results were not scored positively or negatively, but the parameters of each test were compared between evaluators and OP measurements. Prior to the test, the participants were given the opportunity to try both movement tasks with their right and left lower limbs once. Two images were then displayed on a screen in front of the participant. The first slide showed the initial position of the movement, while the second showed the final position of the movement.

### Motion capture setup

For video recording, we purposefully chose a regular RGB camera: a Logitech C920 webcam with a resolution of 1280 x 720 and a frame rate of 30 frames per second, and h264 compression. In order to prove that we could make such measurements with an ordinary camera. We set this webcam on a tripod at a height of 130 cm to the top edge, and at a distance of 4m from the subject.

The camera was pointed perpendicular to the subject so that it would capture motion in the frontal plane. The green screen was placed 0.5 m behind the subject, leaving the distance between the camera and the screen at 4.5 m. Thereby we had the maximum chance of picking up body segments. For our study, we use a green screen in the background only for future use of the video dataset with systems operating on a different principle. The models used by OpenPose are trained in real-life environments. Therefore, the use of the system can also be in an environment with any background provided that there are no other people in the image that the system can also detect.

**Fig 2:**
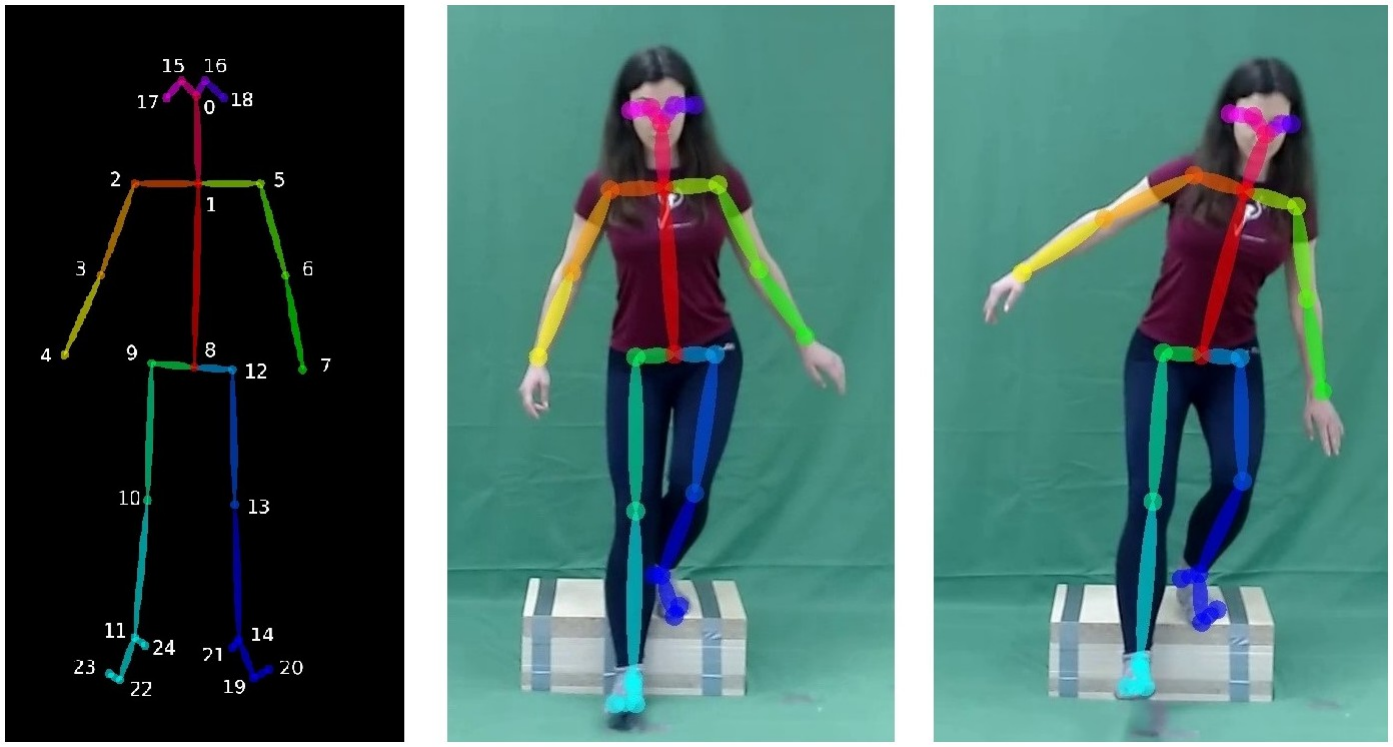
In the left part you can see the 25 keypoints (landmarks) model of the OpenPose. In the middle part is an image with the skeleton rendered while performing the functional test without any error. In the right part, the loss of balance error is clearly visible.

We instructed the subject to perform the aforementioned functional tests as described in the previous section. The Step Down Test was performed on a 20 cm step. Before the test, the subject had the opportunity to try both movements. They were shown two images showing the start and finish positions of both tests for ease of comprehension. Following this, the actual tests were conducted in the order of SLST, followed by SDT. The recorded videos had a length of approximately 15 seconds. A frame of the record can be seen on the right side of the Fig 2.

### Expert visual analysis

Our study utilizes the proficient judgment of three physical therapists (PTs), each holding a Master’s degree in physiotherapy and a practical experience of five years at the time of the study. These experts carried out an Expert Visual Analysis from a vantage point approximately 4.5 meters behind the recording camera, mirroring the camera’s angle to scrutinize the participant’s execution of the Forward-Step-Down-Test (SDT).

The selected configuration mirrors an approach supported by an analogous study from the Israeli Physical Therapy Society [29]. This study established that a rater’s familiarity with the SDT significantly bolsters the agreement rate, thereby emphasizing the critical role that familiarity plays in ensuring the reliability of the test results. Moreover, the study found that the level of work experience did not influence the agreement rate. These key findings reinforce the reliability and validity of our use of the SDT as an evaluation tool, enhancing the credibility of our methodology and the anticipated outcomes of our study.

Each PT independently evaluated the presence of deviations from correct movement execution using an electronic questionnaire and a binary rating scale. The PTs’ scores were evaluated for their inter-rater reliability using Fleiss’s Kappa [30].

As a result, we obtained an expert evaluation, which served as a reference. We used this information for categorization in our classification algorithms. Although we may not obtain precise biomechanical motion data, we can still develop an expert semi-objective reference standard based on subjective data acquired in this manner.

However, it is essential to note the potential limitations of this approach. The binary rating scale, while offering simplicity, may not capture the nuanced differences in movement deviations. Moreover, the validity of this scale is predicated on the expertise and subjective judgment of the PTs. Future studies may benefit from using a more detailed rating scale or incorporating additional methods to enhance the validity and reliability of the visual analysis.

### Signal processing and signal extraction

Each of one subject’s measurements resulted in a single video file containing both tests. In order to minimize distractions, the video image was further cropped to show only the subject and the green screen behind him. With OP, we were able to extract spatiotemporal information from the video files.

OP extracted a human segment model from each frame. The collection of human skeletons is used to illustrate human movement in the video. We grouped the skeletons into a table with a row for each skeleton. The skeleton is anatomically defined by a vector, in which each vector component represents the position of a vertex in the skeleton. Fig 2 illustrates the two-dimensional Cartesian coordinates of a skeleton. The 25 key points of the model are automatically determined by OP. In our previous work, we show which anatomical points correspond to the points in the 25 landmarks model [31].

Among the detected landmarks, we calculate a signal of the range of motion in three variations. Angles and distances are calculated from the created segmental body model, which is based on identified landmarks. Custom-made software programmed in Python was used to calculate angles and distances.

#### Joint angles

Joint angles were calculated as the angle of three points in 2D space. The points correspond to the OP landmark model, please see Fig 2. For example, the angle between the R. acromion, end of the clavicle (collar bone) top of the shoulder (A—2), R. lateral epicondyle of humerus, lateral epicondyle of the humerus, outside of the elbow (B—3) and R. styloid process of the radius, wrist on the thumb side (C—4), see Eq 1.

$$\begin{array}{c} ∢ABC=\text{arccos}\frac{\vec{BA}\cdot \vec{BC}}{\left|BA||BC\right|} \end{array}$$ (1)

#### Keypoint-pair orientation

The Keypoint-pair orientation between two points and the horizontal of the image was calculated. The camera was in a horizontal position. Before starting the measurement, we calibrated the camera position using a laser system we developed [32]. An example of such a calculation was the angle between the center of the pelvis (A—8), the center of the shoulders (B—1) determined by OP, and the horizontal plane. This information gave us information about the tilt of the trunk, see Eq 2.

$$\begin{array}{c} ∡\vec{AB}=\text{arctan}\frac{{\vec{AB}}_{y}}{{\vec{AB}}_{x}} \end{array}$$ (2)

#### Relative distances

The relative distance was the distance in pixels divided by the distance between two points—(A—1) and (B—8). This normalized value can take into account the person’s body height and the distance of the person from the camera, i.e. it eliminates intra-population variations in body height and inaccuracies in the distance between the camera and the body of the measured person. Range of Movement (ROM) was calculated as a difference between the minimum and maximum value of the signal, see Eq 1.

$$\begin{array}{c} {\left|AB\right|}_{r}=\frac{\left|AB\right|}{|K_{1}K_{8}|} \end{array}$$ (3)

#### Feature definition

Based on the definition of functional tests [2], we selected specific elements to describe the correctness of execution, please see Table 1. These key points correspond to the anatomical points, please see study [31].

**Table 1:** List of selected ranges of movements(ROM), from which features were then counted.

Range of Movement—Feature name	Category	Selected keypoints
Hips	Keypoint-pair orientation	(9,12)
BothShoulders	Keypoint-pair orientation	(2,5)
Spine	Keypoint-pair orientation	(1,8)
NeckRShoulderRElbow	Joint angle	(1,2,3)
NeckLShoulderLElbow	Joint angle	(1,5,6)
MidHipRHipRKnee	Joint angle	(8,9,10)
MidHipLHipLKnee	Joint angle	(8,12,13)
RHipRKneeRAnkle	Joint angle	(9,10,11)
LHipLKneeLAnkle	Joint angle	(12,13,14)
NoseNeckMidHip	Joint angle	(0,1,8)
NeckMidHip	Relative distances	(1,8)
RWristMidHip	Relative distances	(4,7)
RWristLWrist	Relative distances	(4,8)
MidHipLSmallToe	Relative distances	(8,13)
MidHipLRSmallToe	Relative distances	(8,10)

In this section, we describe signal processing. We used OP for video processing to extract key body points for each time and create a time series. From this time series, we calculated all presented angles and relative distances as signals. We then used these signals to extract feature parameters such as minima, maxima, medians, and means. The resulting data are available in S1 Data in a readable format. This approach allowed us to analyze the subject’s movements and identify specific features that are suitable for further processing by machine learning algorithms.

We are operating under the assumption that if an expert observes a movement, the relevant information is necessarily captured in the video. Although the expert may not see precise angles or measurements, their experience allows them to determine whether or not errors are present in the movement. Therefore, our focus is not on obtaining a precise biomechanical description, but rather on acquiring data that can distinguish between the presence or absence of evaluated parameters, and investigating whether these derived parameters contain information regarding the correctness of execution. To accomplish this, we utilize machine learning algorithms.

### Classification

We have developed a learning system whose function is to answer six research questions by determining whether a specific phenomenon occurred during the execution of the exercise—six binary answers:

1. Loss of balance
2. Gross arm deviation
3. Trunk movement: Forward lean
4. Depth of squat
5. Posture of the hip joint: Drop
6. Overall Impression: Tremor

To train the machine, we utilized a Python library called PyCaret. PyCaret is a high-performance Python library with low code that facilitates the comparison, training, evaluation, tuning, and deployment of machine-learning models. In this library, we are able to evaluate, compare and adjust standard algorithms of different machine learning algorithms in parallel on the basis of a given data set in an efficient and comparative manner.

We choose to utilize AdaBoost, a method of ensemble learning (also called “meta-learning”), to improve our binary classification performance. The AdaBoost algorithm takes an iterative approach to learn from the mistakes of weak classifiers and converted them into stronger ones. AdaBoost is a sequential learning algorithm. Successive models are generated sequentially and their errors are learned by their successors. By giving the mislabeled examples higher weights, this technique exploits the dependency between models. Just as humans learn from their mistakes and do their best to avoid making the same mistakes in the future, the Boosting algorithm attempts to create a stronger learner (predictive model) from the mistakes of several weaker ones. The purpose of boosting is to reduce the bias error that occurs when models are not able to identify relevant trends in the data. AdaBoost (Adaptive Boosting) is a popular boosting technique that aims to combine multiple weak classifiers into a single strong classifier. The definition of a weak classifier is that it performs better than random guessing but is still ineffective at classifying objects. We have implemented the AdaBoost algorithm by using ten poor decision trees, processed sequentially.

For the AdaBoost classifier design in this study, we partitioned the available data into training, validation, and testing sets, allocating 80%, 15%, and 5% respectively. We utilized a Decision Tree Classifier as the base classifier with 50 weak learners employed in the AdaBoost classifier. The learning rate was set to 0.1 and the maximum depth of the decision trees was limited to 3. The feature subset size was determined as the square root of the total number of features and we set the random state to 42 to ensure reproducibility. We initialized the weights of all data points in the training set to be equal, iteratively training a weak learner on the training data, calculating the weighted error of the weak learner on the training set, calculating the weight of the weak learner based on their performance, and updating the weights of the misclassified samples. After the specified number of iterations, the predictions of all weak learners were combined using their weights. Finally, we evaluated the final AdaBoost classifier on the validation and testing data to assess its performance. The data partitioning and parameter details allow other researchers to replicate the experimental setup and enhance the reproducibility of the results.

## Results

The results section is divided into two logical units. First, we assess the agreement of individual PTs, which we express using Fleis’s kappa. Based on this evaluation, we can proceed to the next section and perform classification on the groups where the agreement, respectively value of Fleis’s Kappa was greater than 0.41. This value is referred to in the literature as Moderate agreement [30]. However, most of the groups achieved much higher values, see Table 2.

**Table 2:** Agreement of the expert ratings of the three physiotherapists. The agreement is expressed by Fleis’s Kappa coefficient. In the literature [30], a kappa greater than 0.41 is considered sufficient. The frequency for each of the binary categories is given in parentheses after this value. The number of subjects who did the given error is labeled as FAULTY and NON-FAULTY respectively. Values subjected to classification are in bold.

Functional test parameters	SDT [kappa (F,NF)]	SLST [kappa (F,NF)]
Overall Impression: Loss of balance	**0,48 (12,34)**	0,56 (8,38)
Overall Impression: Gross arm deviation	**0,58 (8,38)**	0,73 (4,42)
Overall Impression: Disruption of smooth movement	0,37 (7,39)	**0,49 (6,40)**
Overall Impression: Tremor	0,68 (6,40)	**0,59 (10,36)**
Overall Impression: Depth of squat	**0,86 (8,38)**	0,95 (0,46)
Trunk movement: Forward lean	**0,68 (9,37)**	0,95 (2,44)
Trunk movement: Lateroflexion	0,44 (5,41)	0,65 (2,44)
Trunk movement Lateral rotation	0,87 (0,46)	0,93 (3,43)
Posture of the pelvis: Anteversion	1,00 (0,46)	1,00 (0,46)
Posture of the pelvis: Retroversion	1,00 (0,46)	1,00 (0,46)
Posture of the hip joint: Drop	**0,45 (32,14)**	0,84 (5,41)
Posture of the hip joint: Shift	0,24 (9,37)	0,92 (0,46)
Lower limbs: Hip joint, Adduction, internal rotation	0,15(27,19)	0,13 (30,16)
Lower limbs: Knee-joint, valgosity	0,27 (20,26)	0,07(22,24
Lower limbs: Knee-joint, varosity	1,00 (0,46)	1,00 (0,46)

### Agreement of expert visual analysis

In Section, we described the 15 categories that the three independent PTs evaluated. Table 2 shows Fleis’s kappa and the size of each group. Some errors were not observed among the selected participants at all. Such groups could not then be subject to classification. Groups that were suitable for classification are highlighted in bold in the Table 2.

By using the Kappa coefficient to assess agreement between raters, we established a reference standard for classification that divides the data into those that can be used—those with high agreement—and those that cannot be used due to insufficient agreement among the experts. We followed standard methods to ensure the validity and reliability of our approach. Specifically, we used well-established procedures for data collection and analysis, and we implemented rigorous quality control measures to ensure that the results were accurate and reproducible. Overall, our approach provides a robust framework for classifying data and enables us to identify reliable and valid results for further analysis.

### Results of classification

Our results show that if two or more raters agree on the presence of error, and there is a sufficient amount of measured data with and without error, our method based on a single 2D camera produces results comparable to the raters themselves. In the event that even three physiotherapists do not agree on the correct result, then it is likely that the gold standard for machine learning methods cannot be established. Due to the fact that our experts were not in agreement on all measurement categories, we were forced to exclude some of the categories from the evaluation. As a consequence, the experts’ assessment is still a subjective view and can differ depending on their life experience and the weight they give to individual errors.

For classification, we selected only the datasets fulfilling the conditions of kappa coefficient and minimum sample size, see Table 2. These were two separate datasets. Five responses (functional test parameters) to the first dataset (SDT), which contained 15 independent variables, met the conditions. The second data set (SLST) contained 15 independent variables and 2 responses from our panel of expert examiners. We calculated a confusion matrix for each dependent variable.

Our focus was on sensitivity and specificity. It was quite difficult to find subjects who did the movement incorrectly in our sample since only healthy people were included in our sample, according to our experts. In the end, our database is imbalanced because we have included only healthy subjects, the majority of the results showed that the patients were able to perform the exercises correctly. Therefore, we have implemented conventional methods in order to deal with the data imbalance. In this article, we wish to emphasize that since the purpose of the article is to prove the applicability of the method, we have chosen to present a number of cases in which a balance can be illustrated in the analysis. We are therefore very concerned with the sensitivity and specificity of our results in the results section. According to these two metrics, it can be seen that the data we finally used is balanced on the one hand, and on the other hand, they show that our method is able to detect any error by the subject with an average probability of 0.68 (Sensitivity) and that its prediction reliability (i.e. error reporting) is 0.8 (Specificity). The results are displayed in the Table 3.

**Table 3:** Summary of the AdaBoost classification for the two exercises SDT and SLST. In the case of the SDT, it presents five dependent outputs, while for the SLST, two variables are dependent. Dependent variables consist of binary classifications. The quality of classification is determined by the accuracy, specificity, and sensitivity of the classification. The sample is 46, for details, see Table 2.

Functional test parameters (N = 46)	Specif.	Sensit.	Accuracy
**Step down Test (SDT)**			
Overall Impression: Lost of balance	0.95	0.5	0.86
Overall Impression: Gross arm deviation	0.88	0.75	0.86
Trunk movement: Forward lean	0.55	0.67	0.57
Overall Impression: Depth of squat	0.83	0.4	0.75
Posture of the hip joint: Drop	0.38	0.9	0.75
**Single Leg Squat Test (SLST)**			
Overall Impression: Lost of balance	0.92	0.5	0.86
Overall Impression: Tremor	0.92	0	0.82
**Average values**	**0.80**	**0.68**	**0.77**

We used the AdaBoost Classification algorithm for our data analysis, which is a powerful ensemble learning tool for binary or multiclass classification problems. It integrates many weak classifiers to construct a robust classifier that provides accurate and exhaustive data analysis insights. Our approach trained weak classifiers, such as decision trees, on the weighted training set at each iteration to reduce classification error. We adjusted the instance weights by assigning incorrectly classified cases with greater weights and correctly classified instances with smaller weights, ensuring that subsequent weak classifiers focused on increasingly challenging events. The robust classifier produced predictions by weighing the votes of all weak classifiers, with the final forecast based on the class with the highest weighted votes. The AdaBoost algorithm is effective at delivering precise data analysis insights by leveraging the skills of several weak classifiers and focusing on difficult situations during training, resulting in a generalizable model for new, unobserved data.

## Discussion

The aim of our research was to explore the feasibility of using a camera-based approach to automate the standard examination of two functional tests: the Step-down Test (SDT) and the Single-Leg-Stance Test (SLST). A common practice for evaluating these tests is also visual analysis [5], done by experts. According to previous studies, 2D and 3D retroreflective marker analysis [4] is capable of achieving the same results in the frontal plane. In related research, Wouter [33] at. col. uses marker-based 2D analysis to evaluate functional tests of range-of-motion. Remedios et. col [25] compare the absolute differences between the 2D markerless analysis and the 3D marker analysis of the functional load lifting test. In their study, the 2D analysis shows significant bias for the peak values of the ranges of motion. This study records motion in the sagittal plane, which performs worse in detection compared to the frontal plane [23]. In our study, we measure only the frontal plane. In our case, we build a model that does not evaluate the performance using absolute values, but a model that evaluates according to the experts’ responses. Thus, it is not essential for us to obtain absolute values of the angles, but the derived values of the 2D model will suffice. This is where our approach is unique and we can afford to compensate for any inaccuracies of the 2D markerless system. This brings an innovative approach that combines modern image processing techniques with expert knowledge. Our interdisciplinary solution enables the transfer of knowledge and experience into automated processes. The result is a comprehensive subject motion analysis that can be used to improve outcomes in clinical applications. Our method brings new possibilities for the diagnosis and treatment of movement disorders and can serve as a supportive tool for physiotherapy practice and other disciplines using movement analysis.

Based on the results of our study, we believe that the design of an assistance system based on our approach is a promising area for future work. Further data for learning and expert evaluation will be necessary to improve the system. This is a significant challenge that requires an interdisciplinary approach and close collaboration with experts from different fields. However, the relatively low cost and scalability of such a system allow the development process to be accelerated and deployed in many places simultaneously.

In the next phase of our research, we plan to strengthen our expert base and prepare our software for mass subjective evaluation. This will improve the whole system and involve more participants and evaluators. We plan to enable remote expert evaluation from video recordings, which will lead to a more robust evaluation of exercise execution.

Overall, our goal is to create a system that can aid in the diagnosis and treatment of movement disorders while serving as a support tool for physiotherapy and other disciplines that use movement analysis. We believe that our interdisciplinary approach and collaboration with experts will lead to significant advances in this field, allowing us to meet the challenges and accelerate the development of an effective assistive system.

## Conclusion

Traditionally, functional tests have been evaluated either visually by experts or through expensive automated systems that require human interaction. However, both of these options come with significant costs in terms of time, money, and resources. To address this issue, we propose a novel and cost-effective solution that combines modern computer vision techniques with deep learning algorithms and the expertise of physiotherapists. Our method offers a more accurate and efficient way to evaluate functional tests, without the need for costly equipment or extensive human involvement. Our approach can detect errors when performing functional tests, allowing for a more comprehensive assessment of performance quality. Further research will allow us to determine the weighting of these parameters to accurately evaluate the overall quality of performance. Our proposed classifier has a high level of accuracy (0.77 on average) due to the heterogeneous data with and without exercise errors and reliable agreement between physiotherapists. Our findings are very encouraging regarding the feasibility of the camera system for use in the home environment. However, we do not believe that these methods can completely replace the work of physiotherapists. Rather, we consider these methods to be useful complementary tools to physiotherapy. We believe that through further research and collaboration with experts in the field, our approach can bring significant advances in the diagnosis and treatment of movement disorders. This study’s contribution to physical therapy lies in demonstrating the effectiveness of a simple and cost-effective camera-based system combined with machine learning algorithms to evaluate functional test performance. This approach has the potential to significantly increase efficiency and reduce the cost of conventional test measurement and evaluation, expand the range of assessment tools available to physiotherapists, and potentially improve the accuracy and reliability of their evaluations.

## Supporting information

S1 Data Comprehensive dataset of extracted movement keypoints and features. This dataset includes all the extracted keypoints of movements and the associated features and statistics, which were used for the analyses presented in the main manuscript.
(ZIP)
